# Expired Patents

**Published:** 1906-12

**Authors:** 


### EXPIRED PATENTS.

Oct. 22, 1906.

413,376, E. L. Starr, Phila., Pa. Method of Lining Dental Plates with gold.

November 5, 1906.

414,311, H. Haussman, Chicago, Ill. Veterinary Incisor Cutter.

414,353, W. E. Wells, New York, N. Y. Dental Mallet.

November 12, 1906.

412,995, C. M. Richmond, New York, N. Y. Artificial tooth crown.

November 19, 1906.

415,307, T. J. Carrick, Baltimore, Md. Dental Chair.

415,495, B. S. Byrnes, Memphis, Tenn. Dental plugger.

415,594, G. M. Weirich, Phila., Pa. Dental Impression cup.

The above list of patents are furnished by Davis & Davis, solicitors of American and Foreign patents, Washington, D. C., and St. Paul Building, New York City.

